# Mortality from malignant neoplasms at home and in hospitals in Brazil, 2002-2022: sociodemographic characteristics and temporal trends

**DOI:** 10.1590/1980-549720250021

**Published:** 2025-05-02

**Authors:** Patrícia Chatalov Ferreira, Beatriz Jorge Oliveira Gomes, Glaúcia Maria Canato, Eloah Boska Mantovani, Lucas Vinícius de Lima, Gabriel Pavinati, Iven Giovanna Trindade Lino, Sonia Silva Marcon

**Affiliations:** I Universidade Estadual de Maringá, Graduate Program in Nursing – Maringá (PR), Brazil.; II Universidade Estadual de Maringá, Department of Nursing – Maringá (PR), Brazil.; III Secretaria Municipal de Saúde, Planning Management – Maringá (PR), Brazil.

**Keywords:** Neoplasms, Mortality, Hospital mortality, Hospice care, Time series studies

## Abstract

**Objective:**

To analyze the sociodemographic characteristics and temporal trends of deaths due to malignant neoplasms in Brazil, according to whether they occurred at home or in a hospital, from 2002 to 2022.

**Methods:**

This is a descriptive and ecological study analyzing data on cancer deaths from the Brazilian Mortality Information System. The analysis included descriptive measures, mortality rates, and trends based on joinpoint regression of sociodemographic variables, according to the place of death, as well as associations with the occurrence at home or in hospitals.

**Results:**

We analyzed a total of 3,696,553 cancer deaths in Brazil, of which 82.5% occurred in hospitals. The variables positively associated with deaths at home were: men, age between 70 and 79 years and 80 years or older, mixed-race and Indigenous ethnicity, no formal education and one to three years of formal education, and widowed or other marital status. The Northeast and South regions had the highest rates of home mortality, while the Southeast and South regions led in hospital mortality. There was an increasing trend for both deaths occurring at home and those in hospitals nationwide. In the evaluation of home deaths, an increasing trend was observed in nine states and in the Federal District. Regarding hospital deaths, all Brazilian states showed an increasing trend.

**Conclusion:**

The factors that influence the place of death for cancer patients are complex and include support network, access, culture, and the use of healthcare services. Targeted actions for more vulnerable populations and locations are necessary to reverse the growing trend of this condition.

## INTRODUCTION

The place of death is an important indicator in the evaluation of the quality of end-of-life care^1,2,3,4,5,6^. One of the main conditions that require care in this period is malignant neoplasm, which represents one of the leading causes of death in the world^7^. In 2022, there were 20 million new cases and 9.7 million deaths from the disease^8^. In Brazil, it is expected that between 2023 and 2025 there will be approximately 704 thousand cases of cancer per year, 70% of which are concentrated in the South and Southeast regions^9^.

In some cultures, such as in Brazil, death and the place of its occurrence remain a little discussed topic, mainly because of the prevalence of a curative care model marked by the hospitalization of death^10,11^. Several factors influence decision-making about the place of death, such as: culture^1,11,12,13^, presence or absence of palliative care^6,13^, sociodemographic and clinical characteristics of the patient and programmatic aspects of health centers^6,11^, and access to these services^1,11^.

Considering the territorial extension of Brazil, the cultural diversity, and the different social, economic, and health conditions among the population, which mostly depends exclusively on free access to the Brazilian Unified Health System (SUS), there is a need to understand the realities of end-of-life care and the local and regional inequalities that affect deaths from malignant neoplasia in the country. This information can support end-of-life care planning^10,14^.

Authors of a national study analyzing 3,677,415 cancer deaths showed that 82.3% of them occurred in hospitals, whereas home deaths were more frequent in the Northeast (30.2%) and North (24.8%) regions; there was an inverse correlation between home deaths and regional development^14^. Despite the initial drop, home deaths increased again, especially among Indigenous peoples, individuals without formal education, and residents of the North, Northeast, and Midwest regions^14^.

Conversely, in Spain, researchers showed that people with malignant neoplasms who have a higher level of education are more likely to die at home, possibly due to better access to services and knowledge of palliative care^15^. This reality is also observed in developed countries, such as the United States of America^16^ and Israel^7^, in which increasing trends in cancer deaths at home, as well as decreasing trends in hospitals, were identified.

There are few studies on the aspects that influence the place of death of people with cancer in low- and middle-income countries^17,18^ such as Brazil^10,14,19^. Given this gap, by exploring the trends and causes associated with the place of death, it is possible to contribute to the (re)targeting of public policies in an appropriate way^4,5,20^. Moreover, it enables the instrumentalization of healthcare professionals, seeking to develop a more assertive and focused care to people with advanced cancer^20^.

In addition, the Brazilian regions face different economic and programmatic realities. For instance, the lower availability of qualified hospital units and the difficulty in accessing these services, especially during the course of treatment, may influence the occurrence of deaths due to neoplasms at home. Cultural and educational aspects can also guide this outcome, as the personal decision can be sovereign in choosing the place of death.

Taking this into consideration, in order to evaluate the epidemiological and temporal panorama of deaths due to malignant neoplasms in Brazil, especially with regard to the scenario of occurrence for the moment of finitude of life in the last two decades, the objective of this study was to analyze the sociodemographic characteristics and temporal trends of deaths from malignant neoplasms in Brazil, according to whether they occurred at home or in a hospital, from 2002 to 2022.

## METHODS

### Study design and context

This is a descriptive and ecological study whose units of analysis were deaths from neoplasms and Brazilian states. According to census data from 2022, Brazil has a population of 203,080,756 inhabitants and a territorial area of 8,510,417.771 km^2^, subdivided into 26 states and the Federal District^21^. The estimated gross domestic product (GDP) *per capita* for 2021 was BRL 42,247.52^21^, with a Gini index varying around 0.52 that same year^22^.

### Study period and data source

Death records between 2002 and 2022 were considered in the Brazilian Mortality Information System (*Sistema de Informação sobre Mortalidade* – SIM), accessed on April 30, 2024 via the Department of Informatics of the Brazilian Unified Health System (*Departamento de Informática do Sistema Único de Saúde* – DATASUS). The definition of the study period (2002–2022) is due to fact that, in 2002, the National Program for Pain Assistance and Palliative Care (*Programa Nacional de Assistência à Dor e Cuidados Paliativos*) was established, and 2022 was the last year with mortality data available in the SIM on the date of access to DATASUS.

Moreover, data from the Brazilian population were considered, through the study on population estimates (by municipality, sex, and age) for the period from 2000 to 2021, carried out by the Department of Epidemiological Analysis and Surveillance of Noncommunicable Diseases (*Departamento de Análise Epidemiológica e Vigilância de Doenças Não Transmissíveis* – DAENT) of the Brazilian Ministry of Health^23^. In 2022, as census data were still unavailable in DATASUS, the population quantity estimated in the study for 2021 was used.

### Study population and selection criteria

Deaths from malignant neoplasms of people aged 20 years or over, with codes C00 to C97 from the International Statistical Classification of Diseases and Related Health Problems (10th edition), were considered. The data were accessed according to the individual’s state of residence and the year of death. Deaths occurring in “another health facility,” “public highways,” and “others” were excluded because they were not places of interest to the study.

### Variables

The considered variables were: sex (men; women; ignored); age group, in years (20 to 29; 30 to 39; 40 to 49; 50 to 59; 60 to 69; 70 to 79; 80 or over); ethnicity/skin color (white; Black; Asian; mixed-race; Indigenous; ignored); time of formal study, in years (illiterate; one to three; four to seven; eight to 11; 12 or more; ignored); marital status (single; married; widowed; separated; other; ignored); and year of death (2002 to 2022). In addition, stratification was performed by state and regions (North, Northeast, South, Southeast, and Midwest).

### Data processing and analysis

Initially, the sociodemographic variables were described through measures of absolute (N/n) and relative (%) frequency, according to the place of death (home; hospital). In order to compare the difference in the proportions between the variables, Pearson’s χ^2^ test was applied, considering a 5% significance level (p<0.05). The analysis of the adjusted residuals was used for guiding the association, being positive (+) or negative (-) according to values greater than ±1.96^24^.

The annual mortality rates were also estimated, dividing the number of deaths by the resident population, in the same place and period, and multiplying the result by 100 thousand. In the variables “sex” and “age group,” the denominator population corresponded to that estimated with the same demographic characteristics; for the place of residence, the estimated data for the respective region/state were also considered. These analyses were carried out in the Statistical Package for the Social Sciences Statistics, version 25.

Based on these rates, the average values every three years (2002–2004; 2005–2007; 2008–2010; 2011–2013; 2014–2016; 2017–2019; 2020–2022) were presented, allowing the evaluation of their change over the period. With the annual rates, the trend analysis was carried out by joinpoint regression models^25^, stratified by the sociodemographic and residence variables, according to the place of occurrence. The analysis was performed with the aid of the Joinpoint Regression Program, version 5.0.2.

The annual mortality rates from neoplasms, transformed by natural logarithmic function (ln), were considered as the dependent variable (y); and the calendar years of the period, as the independent variable (x)^25^. The log-linear models [ln(y)=x’beta+error], estimated via grid research, were adjusted by the standard errors of the mortality coefficients, calculated according to the recommendations of the literature, and by the first-order autocorrelation estimated from the data^25^.

For the final models, defined by the lowest value of the weighted Bayesian Information Criterion, annual percent changes (APC) and their 95% confidence intervals (95%CI) were estimated. Positive/negative APC indicated the increasing/decreasing trend of mortality rates, respectively, when their 95%CIs were different from the null value (0). APC whose 95%CI contained the null value were interpreted as stationary trend^25^.

### Ethical aspects

In this research, anonymous and public domain data were used, whose available information is in aggregated format and which can be accessed by any citizen/researcher via the DATASUS website (https://datasus.saude.gov.br/). Therefore, as recommended by Resolution No. 674, of May 6, 2022, of the Brazilian National Health Council, there was no need for submission to or consideration by the Research Ethics Committee.

## RESULTS

There were 3,696,553 cancer deaths in Brazil. In the comparison between the profiles of deaths at home and in the hospital, we observed a significant association for all variables. In the analysis of adjusted residuals, the following variables were positively associated with the occurrence at home: men; age between 70 and 79 years and 80 years or over; mixed-race and Indigenous ethnicity/skin color; illiteracy and time of formal study between one and three years; and widowed or other marital status (Table 1).

**Table 1:** Descriptive measures of the sociodemographic characteristics of people with a record of death from cancer (ICD-10: C00 to C97), according to the place of death (home; hospital), Brazil, 2002 to 2022.

Variable	Total		Home		Hospital		p-value*
	N	%	n	%	N	%	
**Sex**							
Men	1,957,131	52.94	364,648^†^	56.46	1,592,483^‡^	52.20	<0.01
Women	1,739,190	47.05	281,231^‡^	43.54	1,457,959^†^	47.79	
Ignored	232	0.01	26	0.00	206	0.01	
**Age group (years)**							
20 to 29	52,610	1.42	4,299^‡^	0.67	48,311^†^	1.58	<0.01
30 to 39	125,435	3.39	13,237^‡^	2.05	112,198^†^	3.68	
40 to 49	319,710	8.65	40,159^‡^	6.22	279,551^†^	9.16	
50 to 59	658,265	17.81	88,440^‡^	13.69	569,825^†^	18.68	
60 to 69	913,471	24.71	139,632^‡^	21.62	773,839^†^	25.37	
70 to 79	917,319	24.83	174,304^†^	26.99	743,015^‡^	24.36	
80 or over	709,743	19.20	185,834^†^	28.76	523,909^‡^	17.17	
**Ethnicity/skin color**							
White	2,193,085	59.33	346,580^‡^	53.65	1,846,505^†^	60.53	<0.01
Black	256,208	6.93	42,727^‡^	6.62	213,481^†^	7.00	
Asian	25,202	0.68	4,182^‡^	0.65	21,020^†^	0.69	
Mixed-race	1,047,916	28.35	223,868^†^	34.66	824,048^‡^	27.01	
Indigenous	5,240	0.14	1,475^†^	0.23	3,765^‡^	0.12	
Ignored	168,902	4.57	27,073	4.19	141,829	4.65	
**Time of formal study (years)**							
None	452,602	12.24	145,905^†^	22.59	306,697^‡^	10.05	<0.01
One to three	824,143	22.29	157,303^†^	24.35	666,840^‡^	21.86	
Four to seven	745,950	20.18	119,063^‡^	18.43	626,887^†^	20.55	
Eight to 11	544,724	14.74	65,687^‡^	10.17	479,037^†^	15.70	
12 or over	297,626	8.05	35,041^‡^	5.43	262,585^†^	8.61	
Ignored	831,508	22.50	122,906	19.03	708,602	23.23	
**Marital status**							
Single	710,014	19.21	123,415^‡^	19.11	586,599^†^	19.23	<0.01
Married	1,697,139	45.91	288,704^‡^	44.69	1,408,435^†^	46.16	
Widowed	747,045	20.21	152,242^†^	23.57	594,803^‡^	19.50	
Separated	258,057	6.98	33,957^‡^	5.26	224,100^†^	7.35	
Other	81,201	2.20	16,304^†^	2.52	64,897^‡^	2.13	
Ignored	203,097	5.49	31,283	4.84	171,814	5.63	
**Year of death**							
2002–2004	379,148	10.26	73,526	11.38	305,622	10.02	<0.01
2005–2007	434,925	11.77	84,540	13.09	350,385	11.49	
2008–2010	485,961	13.15	87,113	13.49	398,848	13.07	
2011–2013	532,728	14.41	86,564	13.40	446,164	14.63	
2014–2016	582,551	15.76	89,474	13.85	493,077	16.16	
2017–2019	633,165	17.13	99,603	15.42	533,562	17.49	
2020–2022	648,075	17.52	125,085^‡^	19.37	522,990^†^	17.14	
**Total**	**3,696,553**	**100.00**	**645,905**	**100.00**	**3,050,648**	**100.00**	**N/A**

ICD-10: International Statistical Classification of Diseases and Related Health Problems (10th edition); N/A: not applicable;

*Pearson’s χ^2^ test (excluding ignored cases);

^†^positively significant association;

^‡^negatively significant association.

According to the historical series of deaths in the study period, there are different patterns between the variables. Overall, the average mortality rates in the period due to malignant neoplasms in hospitals were higher than those at home, with the following characteristics standing out: men (87.19/100 thousand), age 80 years or over (822.87/100 thousand), white (46.78/100 thousand), one to three years of formal study (20.57/100 thousand), and married marital status (35.46/100 thousand) (Supplementary Material 1: https://doi.org/10.17632/fbcr95km4d.1 ).

The Northeast and South regions had the highest rates of home mortality, reaching 21.25/100 thousand in 2022 and 20.64/100 thousand in 2007, respectively. In 2022, the highest average hospital mortality rates were observed in the Southeast (79.72/100 thousand) and, again, in the South (90.26/100 thousand) regions. In addition, the states with the highest values were Ceará (33.49/100 thousand), at home, and Rio Grande do Sul (118.87/100 thousand), in hospitals (Supplementary Material 2: https://doi.org/10.17632/fbcr95km4d.1 ).

We observed a more pronounced increasing trend for home deaths among men and hospital deaths among women. With regard to the age group, the trend was mostly stationary. Regarding ethnicity/skin color and marital status, we observed increases in the historical series of all hospital deaths. Regarding level of education, we verified an increase in all stratifications, except for people with one to three years of formal study who died in hospitals (Table 2).

**Table 2:** Temporal trend of gross mortality rates (per 100 thousand inhabitants) from cancer (ICD-10: C00 to C97), according to the place of death (home; hospital) and sociodemographic characteristics, Brazil, 2002 to 2022.

Variable	Home			Hospital		
	APC	95%CI	Trend	APC	95%CI	Trend
**Sex**						
Men	1.72	0.76–2.79	Increasing	1.93	1.30–2.78	Increasing
Women	1.53	0.12–3.00	Increasing	2.54	2.00–3.25	Increasing
**Age group (years)**						
20 to 29	-1.49	-3.25–0.18	Stationary	0.37	-0.02–0.77	Stationary
30 to 39	-1.65	-2.54–-0.71	Decreasing	0.33	0.06–0.64	Increasing
40 to 49	-2.02	-3.22–-0.92	Decreasing	-0.67	-1.04–-0.29	Decreasing
50 to 59	-1.29	-2.40–-0.11	Decreasing	-0.58	-1.55–0.35	Stationary
60 to 69	-0.88	-2.43–0.69	Stationary	-0.00	-0.48–0.58	Stationary
70 to 79	-0,74	-1.82–0.39	Stationary	0.11	-0.56–0.92	Stationary
80 or over	-0.57	-1.54–0.63	Stationary	0.10	-0.71–1.19	Stationary
**Ethnicity/skin color**						
White	0.90	-0.71–2.24	Stationary	1.47	1.01–1.99	Increasing
Black	3.11	1.82–4.42	Increasing	3.38	2.96–3.84	Increasing
Asian	0.63	-0.65–2.08	Stationary	2.05	1.65–2.47	Increasing
Mixed-race	3.64	2.44–5.09	Increasing	4.96	3.86–6.49	Increasing
Indigenous	1.25	0.87–1.56	Increasing	2.45	2.09–2.86	Increasing
**Time of formal study (years)**						
None	1.41	0.34–2.52	Increasing	1.73	0.67–3.07	Increasing
One to three	1.10	0.66–1.55	Increasing	1.96	-1.60–4.35	Stationary
Four to seven	4.06	2.59–5.52	Increasing	3.38	2.55–4.51	Increasing
Eight to 11	7.59	6.21–9.32	Increasing	7.86	7.24–8.78	Increasing
12 or over	4.29	1.85–6.70	Increasing	4.78	4.30–5.36	Increasing
**Marital status**						
Single	2.12	0.67–3.74	Increasing	3.11	2.68–3.59	Increasing
Married	0.34	-0.81–1.38	Stationary	1.19	0.65–1.81	Increasing
Widowed	1.49	0.37–2.50	Increasing	1.81	1.20–2.50	Increasing
Separated	6.50	5.25–8.47	Increasing	5.20	4.38–6.52	Increasing
Other	7.76	4.71–13.69	Increasing	7.01	6.74–12.42	Increasing

ICD-10: International Statistical Classification of Diseases and Related Health Problems (10th edition); APC: annual percent change; 95%CI: 95% confidence interval (lower limit; upper limit).

There was an increasing trend nationwide for both home and hospital deaths; the same was observed for the North and Northeast regions. In the evaluation of home deaths, we observed an increasing trend in nine states and in the Federal District, with Bahia being the most accentuated. Regarding hospital deaths, all states showed an increasing trend, especially Piauí (Table 3).

**Table 3:** Temporal trend of gross mortality rates (per 100 thousand inhabitants) from cancer (ICD-10: C00 to C97), according to the place of death (home; hospital) and residence location, Brazil, 2002 to 2022.

Variable	Home			Hospital		
	APC	95%CI	Trend	APC	95%CI	Trend
**North Region**	1.88	1.15–2.75	Increasing	3.95	2.91–5.53	Increasing
Rondônia	2.49	0.02–5.64	Increasing	4.11	3.09–5.47	Increasing
Acre	1.90	-0.25–4.81	Stationary	2.82	1.30–5.10	Increasing
Amazonas	1.63	0.83–2.63	Increasing	2.99	1.56–4.92	Increasing
Roraima	1.40	-0.64–4.39	Stationary	3.41	2.36–4.86	Increasing
Pará	2.28	1.48–3.24	Increasing	4.38	3.61–5.46	Increasing
Amapá	-1.05	-4.02–2.43	Stationary	4.50	2.83–7.32	Increasing
Tocantins	0.88	-0.94–2.95	Stationary	4.60	3.13–6.86	Increasing
**Northeast Region**	2.31	0.81–4.33	Increasing	4.02	3.20–5.33	Increasing
Maranhão	2.27	-0.53–6.70	Stationary	5.28	4.10–7.30	Increasing
Piauí	2.09	0.54–4.11	Increasing	5.65	4.23–8.38	Increasing
Ceará	1.16	0.03–2.45	Increasing	4.05	3.13–5.39	Increasing
Rio Grande do Norte	1.18	-0.89–3.79	Stationary	3.63	2.67–4.99	Increasing
Paraíba	1.09	-1.65–4.94	Stationary	4.93	3.90–6.58	Increasing
Pernambuco	1.21	-0.09–2.75	Stationary	2.61	1.87–3.54	Increasing
Alagoas	1.51	-0.24–3.59	Stationary	5.18	4.39–6.26	Increasing
Sergipe	1.35	-0.11–2.96	Stationary	3.02	1.67–4.86	Increasing
Bahia	4.43	3.02–6.37	Increasing	4.00	2.96–5.32	Increasing
**Southeast Region**	1.18	-0.65–2.87	Stationary	1.57	1.08–2.20	Increasing
Minas Gerais	2.68	1.15–4.40	Increasing	2.76	2.09–3.57	Increasing
Espírito Santo	-0.39	-2.13–1.39	Stationary	2.43	1.49–3.64	Increasing
Rio de Janeiro	-0.09	-1.81–1.58	Stationary	0.83	0.34–1.41	Increasing
São Paulo	1.07	-0.68–2.86	Stationary	1.40	0.96–1.93	Increasing
**South Region**	1.20	-0.47–2.76	Stationary	1.94	1.44–2.58	Increasing
Paraná	0.21	-0.91–1.36	Stationary	2.34	1.70–3.09	Increasing
Santa Catarina	1.68	0.13–3.39	Increasing	2.59	1.97–3.38	Increasing
Rio Grande do Sul	1.98	-0.05–3.91	Stationary	1.52	0.98–2.16	Increasing
**Midwest Region**	1.15	-0.20–2.72	Stationary	2.48	1.80–3.36	Increasing
Mato Grosso do Sul	-0.41	-2.04–1.36	Stationary	2.36	1.71–3.13	Increasing
Mato Grosso	0.66	-1.10–2.88	Stationary	3.07	2.29–4.10	Increasing
Goiás	2.03	0.65–3.82	Increasing	2.71	1.77–3.97	Increasing
Federal District	1.55	0.51–2.77	Increasing	1.38	0.80–2.10	Increasing
**Brazil**	1.58	0.46–2.83	Increasing	2.19	1.62–3.00	Increasing

ICD-10: International Statistical Classification of Diseases and Related Health Problems (10th edition); APC: annual percent change; 95%CI: 95% confidence interval (lower limit; upper limit).

## DISCUSSION

With the stratified trend analysis, in this study we obtained unique and comprehensive results of how sociodemographic characteristics influence the place of death of people with cancer over more than 20 years in Brazil, also considering their geographical and programmatic particularities. Furthermore, we presented trends stratified by sociodemographic and territorial aspects, providing insights into how the disease has affected the population during the evaluated period.

In order to contextualize these findings, studies carried out in different regions of Brazil can contribute to the understanding of the identified trends. In the city of São Paulo (state of São Paulo), individual characteristics, such as women, Black and mixed-race ethnicity/skin color, married/widowed marital status, and high level of education, were associated with a lower chance of home death^17^. Although the results are similar, there are divergences with the results of our research, in which being a widowed was associated with the occurrence at home.

Another relevant factor that can influence the place of death is advanced age. This relationship has already been verified in national^14,17^ and international studies^7,19,20,26^. Older adults in end-of-life care due to advanced cancer are usually cared for at home by formal or informal caregivers, which can largely contribute to their remaining in this environment until the end of life^5,19^. This phenomenon can occur either by the patient’s own choice or by family decision.

Higher occurrence of deaths at home in men has also been evidenced^14,17,20^. This may be related to the fact that women are traditionally more willing to assume the role of caregiver^17^. Although a causal relationship cannot be established in this regard, it is inferred that women could take a more proactive role in the care of the partner/family member with cancer at home, contributing to the increase in deaths in this environment among this population.

In turn, the relation between being a widowed and dying at home can be explained by the absence of a partner who encourages the search for medical care or makes the decision for the person^2,19^. Corroborating this hypothesis, Chinese researchers identified that living with a spouse increases the probability of hospital death by 72%^27^. Therefore, family support can contribute to death occurring at home^19^. Conversely, the lack of availability of family caregivers for palliative care at home makes it difficult^2,28^.

Brazilian researchers identified that, despite the predominance of hospital deaths, most adults with cancer preferred to die at their own home^10^; international studies reinforce this preference^17,29^. However, in addition to the fact that there is not always agreement between patient and caregiver^10^, few patients who wish to die at home have this desire met^2,30^. The actual place of death depends not only on the patient’s clinical status^2,30^, but also on their relationship with the primary caregiver^2^.

Socioeconomic status can have a strong impact on the place of death from neoplasms. In more developed states, hospital mortality remained high, reflecting greater access to hospitals specialized in oncology. Conversely, states such as Bahia and Pará showed increasing trends in home deaths, possibly associated with geographical barriers and lower levels of education, factors that can reduce the search for hospitalizations and favor the permanence at home in end-of-life care.

Furthermore, the cultural, socioeconomic, and healthcare diversity between countries entails different ways of dealing with issues related to death and dying^10,19^. This scenario is also a reality in Brazil, because of its vast territory and the important cultural, social, and economic miscegenation, partly justifying the more accentuated trends in certain places and strata as well as the different mortality rates in the states.

The trends in mortality from neoplasms in the states reflect structural inequalities in the Brazilian health system. On the one hand, São Paulo and Rio Grande do Sul presented high hospital rates, possibly associated with the greater supply of oncological reference units. On the other hand, states in the North and Northeast, such as Maranhão and Piauí, registered increasing trends in home deaths, which may be related to low coverage of specialized services and difficulties in accessing them.

In the last decade, there was a considerable increase in the number of palliative care institutions in Brazil, from 92 services in 2012 to 198 in 2020, corresponding to 0.94 services/million inhabitants^31,32,33^. Although the coverage rate and reach of these services in the country are still incipient, the increase in the number of home deaths may be related to the improvement in access to palliative care due to the increase in the number of available services^18^.

The Family Health Strategy, the Better at Home Program (*Programa Melhor em Casa*), and the National Humanization Policy (*Política Nacional de Humanização*) can also boost the transition from hospital death to home death^34,35,36^. These strategies can provide greater access to palliative care among the population, as well as take into account the singularities and desires of each individual, favoring care that provides quality of life to the remaining days in a more inclusive and comprehensive way.

Nevertheless, authors of a study that analyzed the challenges and possibilities of patients in palliative care returning to their homes found that, in Brazil, the resources made available by SUS for end-of-life care at home are still meagre^35^. This datum must be considered in the interpretation of the findings of the present study, as the highest rates of death due to malignant neoplasm at home may result from the unavailability of hospital services in different locations in the country.

It is worth mentioning the effects of the new coronavirus (COVID-19) pandemic on the results of this research, as it influenced cancer deaths and countless other causes worldwide. In Japan, after 2019, considering all causes, there was an increase in the number of deaths at home and a decrease in hospital deaths, especially among people aged 65 years or over^37^. Factors, such as competition for beds and restricted access to healthcare services, contributed to this change^37,38^.

Although this was not the main focus of our study, such findings may partially explain those of this research during the pandemic period. Nonetheless, it should be noted that, in the type of approach adopted, historical series with long intervals, such as the analyses of about 20 years, tend to be less sensitive to specific fluctuations in a short period of time — such as those related to the specific effects of a recent event like the pandemic.

At the national level, the absence of a program that correctly directs end-of-life care can cause deaths at home due to cancer to be related to the difficulty of access, and not to an individual option. The approval of the National Policy on Palliative Care (*Política Nacional de Cuidados Paliativos*) within the scope of the SUS, in 2023^34^, may favor the development and dissemination of materials aimed at guiding Brazilian healthcare professionals concerning end-of-life care.

Therefore, healthcare professionals, especially nurses, due to their greater proximity to the population, must be prepared to help families and even people with cancer to reflect on the positive and negative aspects of dying at home or in the hospital. The support and advice of a multidisciplinary team are deemed to be decisive in guaranteeing autonomy and in the consequent decision-making of the family/patient in relation to end-of-life care.

The possible limitations of the study are due to the use of secondary data, which may contain incomplete data, filling errors, or underreporting of deaths from malignant neoplasms. As secondary sources are used, we cannot state whether the place of death was a patient’s choice. Another factor that may have interfered with the results of the research was the COVID-19 pandemic, considering that it influenced deaths from neoplasms and numerous other causes at the global level.

Nonetheless, the results of this study contribute to a broader understanding of the relationship between sociodemographic factors and the place of death of cancer patients. We observed that there was a progressive increase in deaths from cancer in hospitals, whereas the increase in deaths at home occurred only in some locations and was influenced by factors such as men, advanced age, mixed-race or Indigenous ethnicity/skin color, low level of education (up to three years of formal study), and widowed marital status.

Thus, the factors that influence the place of death of people with cancer are multifaceted and may be related to the (un)existing support network, the (lack of) access and use of healthcare services, the sociocultural context, and sociodemographic characteristics. Consequently, studies are necessary, including a qualitative approach, to investigate and deepen the aspects that influence the choice of the place of death of people with cancer.

## Data Availability

This research used anonymous and public domain data whose available information is in aggregated format. Therefore, as recommended by Resolution No. 674, of May 6, 2022, of the Brazilian National Health Council, submission to and consideration by the Research Ethics Committee were waived.
