# Supplementary material for: Mortality from malignant neoplasms at home and in hospitals in Brazil, 2002-2022: sociodemographic characteristics and temporal trends
Source: Rev Bras Epidemiol. 2025 May 2;28:e250021. doi: 10.1590/1980-549720250021 (PMC12054985; doi:10.1590/1980-549720250021)
Supplement: Supplementary file 1 [file 1980-5497-rbepid-28-e250021-suppl.pdf]

Tabela Suplementar 1. Taxas brutas de mortalidade (a cada 100.000 habitantes) por neoplasias (CID-10: C00 a C97), segundo o local de ocorrência do óbito (domicílio; hospital), de acordo com as características sociodemográficas da pessoa, Brasil, 2002–2004, 2005–2007, 2008–2010, 2011–2013, 2014–2016, 2017–2019 e 2020–2022.

	Domicílio							Hospital						
**Variável**	**2002–2004**	**2005–2007**	**2008–2010**	**2011–2013**	**2014–2016**	**2017–2019**	**2020–2022**	**2002–2004**	**2005–2007**	**2008–2010**	**2011–2013**	**2014–2016**	**2017–2019**	**2020–2022**
**Sexo**														
Masculino	15,24	17,15	17,38	16,99	17,14	18,50	22,48	61,48	67,76	74,42	80,30	86,06	89,70	84,97
Feminino	11,94	13,05	12,81	12,21	12,27	13,46	16,83	51,41	57,29	63,56	69,87	75,70	81,08	78,99
**Faixa etária**														
20 a 29 anos	0,72	0,71	0,63	0,55	0,47	0,51	0,62	6,34	6,61	6,80	6,78	7,04	6,86	6,69
30 a 39 anos	2,44	2,39	2,14	1,93	1,74	1,79	2,00	16,56	16,80	17,15	17,27	17,80	18,03	17,19
40 a 49 anos	9,30	8,95	8,09	7,14	6,17	6,22	6,92	53,88	53,94	54,19	53,64	51,81	49,73	46,71
50 a 59 anos	25,27	25,18	23,31	20,05	18,72	19,17	21,41	135,92	141,27	144,80	146,33	144,38	137,86	122,70
60 a 69 anos	61,49	61,36	55,06	48,71	45,36	46,12	53,90	281,59	284,42	290,69	293,84	299,76	300,71	272,40
70 a 79 anos	129,54	137,55	126,77	113,90	107,77	105,88	120,39	473,04	508,61	519,76	525,51	529,30	521,90	468,80
80 anos ou mais	293,10	309,57	289,02	264,36	250,67	250,47	289,67	723,75	748,97	774,86	806,91	824,12	811,96	713,99
**Raça/cor**														
Branca	8,35	8,35	8,05	7,59	7,55	8,19	10,25	37,56	40,19	43,01	45,36	47,63	49,33	46,85
Preta	0,81	0,90	0,93	0,98	0,95	1,12	1,46	3,62	4,04	4,55	5,21	5,57	6,21	6,42
Amarela	0,10	0,10	0,09	0,09	0,09	0,10	0,13	0,42	0,43	0,47	0,52	0,54	0,56	0,58
Parda	3,28	4,64	5,13	5,23	5,56	6,13	7,34	10,60	13,48	16,45	19,87	23,51	26,67	26,15
Indígena	0,02	0,03	0,03	0,03	0,04	0,04	0,05	0,05	0,06	0,08	0,09	0,10	0,13	0,13

Variável	Domicílio 2002–2004	2005–2007	2008–2010	2011–2013	2014–2016	2017–2019	2020–2022	Hospital 2002–2004	2005–2007	2008–2010	2011–2013	2014–2016	2017–2019	2020–2022
**Tempo de estudo**														
Nenhum	2,78	3,50	3,51	3,54	3,56	3,64	3,95	5,81	6,08	6,68	7,95	8,43	8,62	7,80
1 a 3 anos	3,38	3,57	3,57	4,00	3,83	3,82	4,27	11,21	11,81	12,88	19,78	20,61	19,13	16,00
4 a 7 anos	2,11	2,36	2,66	2,39	2,60	3,29	4,43	10,14	12,47	15,16	13,80	15,59	18,70	18,77
8 a 11 anos	0,82	0,90	1,06	1,32	1,56	2,07	3,10	4,82	6,23	8,04	10,74	13,40	16,66	19,01
12 anos ou mais	0,67	0,71	0,74	0,65	0,72	0,93	1,41	3,79	4,59	5,66	5,77	6,86	8,13	8,84
**Estado civil**														
Solteiro	2,37	2,91	3,05	2,75	2,72	3,09	3,80	10,07	11,48	13,14	13,73	15,43	16,92	17,18
Casado	6,90	7,40	7,19	6,44	6,26	6,62	7,86	28,92	31,39	33,58	34,52	36,12	37,44	34,61
Viúvo	3,22	3,54	3,53	3,39	3,48	3,75	4,64	11,59	12,77	13,87	14,55	15,46	16,30	15,20
Separado	0,46	0,56	0,66	0,72	0,79	1,00	1,43	2,78	3,64	4,66	5,27	6,19	7,20	7,43
Outro	0,13	0,11	0,03	0,38	0,55	0,64	0,81	0,38	0,25	0,09	1,63	2,33	2,87	2,99

CID-10: Classificação Estatística Internacional de Doenças e Problemas Relacionados à Saúde (10ª edição).

Tabela Suplementar 2. Taxas brutas de mortalidade (a cada 100.000 habitantes) por neoplasias (CID-10: C00 a C97), segundo o local de ocorrência do óbito (domicílio; hospital), de acordo com o local de residência da pessoa, Brasil, 2002–2004, 2005–2007, 2008–2010, 2011–2013, 2014–2016, 2017–2019 e 2020–2022.

	Domicílio							Hospital						
**Variável**	**2002–2004**	**2005–2007**	**2008–2010**	**2011–2013**	**2014–2016**	**2017–2019**	**2020–2022**	**2002–2004**	**2005–2007**	**2008–2010**	**2011–2013**	**2014–2016**	**2017–2019**	**2020–2022**
**Região Norte**	9,86	10,99	11,18	11,10	12,02	12,66	14,71	23,06	26,98	31,36	38,32	43,78	47,77	45,54
Rondônia	10,42	9,45	9,88	9,51	9,46	11,87	16,66	30,10	31,98	40,89	45,95	56,26	61,01	57,51
Acre	5,30	13,54	9,57	9,50	10,27	13,19	11,92	25,09	28,73	30,85	42,43	46,56	49,60	45,16
Amazonas	11,49	13,69	14,05	13,73	13,89	15,28	17,08	25,79	30,68	34,13	43,19	46,57	47,56	43,30
Roraima	14,66	12,42	11,94	9,76	11,72	15,33	17,55	23,70	32,13	30,75	34,92	38,29	47,07	44,34
Pará	7,84	9,35	10,27	10,27	12,12	11,30	12,93	20,38	24,25	28,10	33,58	39,07	44,16	43,43
Amapá	11,77	10,43	5,51	6,01	6,62	7,51	10,18	20,53	20,61	23,27	38,19	41,88	44,10	44,51
Tocantins	16,19	13,52	13,80	14,60	13,33	15,57	18,84	22,53	27,40	35,91	42,29	49,67	54,33	50,74
**Região Nordeste**	13,03	19,96	20,96	20,21	20,49	21,90	24,88	29,32	35,58	41,99	49,11	55,33	61,20	59,73
Maranhão	5,08	14,39	14,61	14,45	15,80	15,88	17,27	15,25	18,96	25,33	31,10	34,90	41,55	41,06
Piauí	17,43	24,72	27,43	27,67	27,73	27,44	31,49	18,17	23,99	31,39	40,43	49,78	54,08	51,27
Ceará	24,64	31,70	32,59	30,45	31,22	34,40	35,71	28,27	36,75	41,54	49,83	55,21	59,50	58,73
Rio Grande do Norte	14,43	22,85	23,80	22,92	23,75	21,63	23,69	35,95	43,15	50,28	58,43	65,05	73,13	68,61
Paraíba	10,63	26,19	29,28	27,58	24,48	22,96	27,13	29,47	37,42	45,28	55,16	64,51	72,72	71,87
Pernambuco	15,25	20,68	19,45	18,57	18,51	19,59	22,50	43,84	50,34	55,76	60,89	67,06	72,21	68,91
Alagoas	10,59	16,90	17,14	16,79	16,80	17,54	18,50	22,00	25,81	29,00	35,71	44,63	51,25	53,51
Sergipe	14,20	19,31	20,36	19,46	18,82	19,54	22,56	30,32	36,86	43,99	48,71	53,55	56,46	52,93
Bahia	8,22	12,55	14,50	14,40	15,08	18,50	23,38	29,72	35,73	43,60	50,96	56,75	63,12	62,26
**Região Sudeste**	12,39	11,64	11,32	10,88	10,82	12,06	15,94	73,22	79,24	85,80	90,42	95,89	99,99	95,32
Minas Gerais	13,46	13,03	13,41	13,28	14,34	16,34	21,62	52,27	61,15	67,84	74,81	81,23	85,26	84,83

Variável	Domicílio 2002–2004	Domicílio 2005–2007	Domicílio 2008–2010	Domicílio 2011–2013	Domicílio 2014–2016	Domicílio 2017–2019	Domicílio 2020–2022	Hospital 2002–2004	Hospital 2005–2007	Hospital 2008–2010	Hospital 2011–2013	Hospital 2014–2016	Hospital 2017–2019	Hospital 2020–2022
Espírito Santo	17,76	17,87	15,77	13,61	13,13	14,39	16,80	55,37	61,44	73,17	81,57	85,64	90,59	86,18
Rio de Janeiro	12,31	11,30	11,61	10,83	9,26	9,49	12,76	89,75	94,00	100,18	103,02	107,44	109,55	101,53
São Paulo	11,46	10,58	9,84	9,53	9,57	10,85	14,45	78,43	83,68	89,83	93,68	99,22	104,02	98,61
**Região Sul**	20,40	19,70	18,86	18,46	18,23	19,76	25,45	82,09	90,02	98,21	106,69	112,56	116,99	113,36
Paraná	25,24	23,70	22,37	22,27	21,94	22,58	27,14	63,83	70,90	79,34	87,00	92,53	97,50	96,92
Santa Catarina	17,85	17,15	17,32	17,65	17,22	17,55	23,99	66,75	72,84	81,84	91,15	97,92	104,58	102,45
Rio Grande do Sul	17,18	17,29	16,33	15,19	15,17	18,32	24,66	107,80	118,02	125,98	135,25	141,35	144,27	136,93
**Região Centro-Oeste**	12,70	11,34	10,97	10,71	11,12	12,46	15,82	46,40	52,22	58,71	64,39	70,89	74,03	70,50
Mato Grosso do Sul	16,56	15,17	13,76	12,47	12,04	13,14	15,82	54,78	63,37	70,79	73,72	80,18	86,60	83,47
Mato Grosso	13,86	13,22	11,49	10,36	10,42	11,50	15,72	35,71	40,02	46,56	53,61	59,66	61,63	60,14
Goiás	12,87	10,74	11,44	11,71	12,79	14,73	18,57	43,96	50,23	56,92	63,62	71,95	73,64	69,68
Distrito Federal	7,06	6,79	6,62	7,12	7,21	7,64	9,51	56,89	60,73	65,75	70,04	72,86	77,67	72,44
**Brasil**	13,56	15,06	15,05	14,55	14,66	15,92	19,60	56,37	62,43	68,89	74,98	80,78	85,30	81,92

CID-10: Classificação Estatística Internacional de Doenças e Problemas Relacionados à Saúde (10ª edição).
