# Supplementary material for: Mortality from malignant neoplasms at home and in hospitals in Brazil, 2002-2022: sociodemographic characteristics and temporal trends
Source: Rev Bras Epidemiol. 2025 May 2;28:e250021. doi: 10.1590/1980-549720250021 (PMC12054985; doi:10.1590/1980-549720250021)
Supplement: Translation

# Translation: Mortalidade por neoplasias malignas em domicílio e hospital no Brasil, 2002–2022: características sociodemográficas e tendências temporais

**DOI:** 10.1590/1980-549720250021.2

**Authors:** Patrícia Chatalov Ferreira, Beatriz Jorge Oliveira Gomes, Glaúcia Maria Canato, Eloah Boska Mantovani, Lucas Vinícius de Lima, Gabriel Pavinati, Iven Giovanna Trindade Lino, Sonia Silva Marcon

**Affiliations:** I Universidade Estadual de Maringá, Programa de Pós-Graduação em Enfermagem – Maringá (PR), Brasil.; II Universidade Estadual de Maringá, Departamento de Enfermagem – Maringá (PR), Brasil.; III Secretaria Municipal de Saúde, Gerência de Planejamento – Maringá (PR), Brasil.

**Keywords:** Neoplasias, Mortalidade, Mortalidade hospitalar, Cuidados paliativos na terminalidade da vida, Estudos de séries temporais

## Abstract

**Objetivo:**

Analisar as características sociodemográficas e as tendências temporais dos óbitos por neoplasias malignas no Brasil, segundo a ocorrência em domicílio ou hospital, no período de 2002 a 2022.

**Métodos:**

Estudo descritivo e ecológico que analisou dados sobre óbitos por neoplasias do Sistema de Informação sobre Mortalidade. A análise incluiu medidas descritivas, taxas de mortalidade e tendências por regressão *joinpoint* de variáveis sociodemográficas, conforme o local de óbito, além de associação com a ocorrência em domicílio ou hospital.

**Resultados:**

Analisaram-se 3.696.553 óbitos por neoplasias no Brasil, dos quais 82,5% ocorreram em hospitais. Associaram-se positivamente à ocorrência em domicílio as variáveis: sexo masculino, idade entre 70 e 79 anos e 80 anos ou mais, raça/cor parda e indígena, ausência de estudo e tempo de estudo entre um e três anos, e estado civil viúvo ou outro. As regiões Nordeste e Sul tiveram as maiores taxas de mortalidade domiciliar, enquanto Sudeste e Sul lideraram na hospitalar. Houve tendência crescente tanto para os óbitos ocorridos em domicílio quanto para aqueles em hospital no país. Na avaliação dos óbitos ocorridos em domicílio, notou-se tendência de incremento em nove estados e no Distrito Federal. No que se refere aos óbitos em hospital, todas as unidades da federação registraram tendência crescente.

**Conclusão:**

Os fatores que influenciam o local de morte de pessoas com câncer são complexos e incluem rede de apoio e acesso e disponibilidade de serviços de saúde. Ações direcionadas a públicos e locais mais vulneráveis são necessárias para reverter o cenário ascendente desse agravo.

## INTRODUÇÃO

O local de morte constitui importante indicador na avaliação da qualidade dos cuidados no fim da vida^1,2,3,4,5,6^. Uma das principais condições que demandam cuidados nesse período é a neoplasia maligna, que representa uma das maiores causas de morte no mundo^7^. Em 2022, ocorreram 20 milhões de novos casos e 9,7 milhões de óbitos pelo agravo^8^. No Brasil, prevê-se que entre 2023 e 2025 surjam aproximadamente 704 mil casos de câncer por ano, sendo 70% deles concentrados nas regiões Sul e Sudeste^9^.

Em algumas culturas, como na do Brasil, a morte e o local de sua ocorrência seguem como tema pouco discutido, sobretudo por causa da prevalência de um modelo assistencial curativista marcado pela hospitalização da morte^10,11^. Diversos fatores influenciam a tomada de decisão acerca do local de morte, tais como: cultura^1,11,12,13^, presença ou não dos cuidados paliativos^6,13^, características sociodemográficas e clínicas do paciente e aspectos programáticos das unidades de saúde^6,11^ e de acesso a esses serviços^1,11^.

Considerando a extensão territorial do Brasil, a diversidade cultural e as díspares condições sociais, econômicas e de saúde entre a população, que em sua maioria depende exclusivamente do acesso gratuito ao Sistema Único de Saúde (SUS), percebe-se a necessidade de compreender as realidades dos cuidados de fim de vida e as desigualdades locorregionais que tangenciam as mortes por neoplasia maligna no país. Essas informações podem subsidiar o planejamento de cuidados de fim da vida^10,14^.

Estudo nacional analisando 3.677.415 óbitos por câncer mostrou que 82,3% deles ocorreram em hospitais, enquanto as mortes domiciliares foram mais frequentes nas regiões Nordeste (30,2%) e Norte (24,8%); houve correlação inversa entre os óbitos domiciliares e o desenvolvimento regional^14^. Apesar da queda inicial, as mortes domiciliares voltaram a crescer, especialmente entre povos originários, pessoas sem educação formal e residentes das regiões Norte, Nordeste e Centro-Oeste^14^.

Em contrapartida, na Espanha, estudo mostrou que pessoas com neoplasias malignas que apresentam nível superior de educação têm maior probabilidade de morrer em casa, possivelmente em função do melhor acesso a serviços e conhecimento sobre os cuidados paliativos^15^. Tal realidade também é visualizada em países desenvolvidos como Estados Unidos da América^16^ e Israel^7^, que identificaram tendências crescentes dos óbitos por câncer em domicílio, bem como decrescentes nos hospitais.

Há poucos estudos sobre os aspectos que influenciam o local de morte de pessoas com câncer em países de baixa e média renda^17,18^, como o Brasil^10,14,19^. Diante dessa lacuna, ao explorar as tendências e as causas associadas ao local do óbito, é possível contribuir com o (re)direcionamento de políticas públicas de maneira adequada^4,5,20^. Mais ainda, permite-se a instrumentalização dos profissionais de saúde, a fim de desenvolver uma assistência mais assertiva e centrada às pessoas com câncer avançado^20^.

Adicionalmente, as regiões do Brasil enfrentam diferentes realidades econômicas e programáticas. Por exemplo, a menor disponibilidade de unidades hospitalares habilitadas e a dificuldade no acesso a esses serviços, especialmente durante o percurso de tratamento, podem influenciar a ocorrência dos óbitos por neoplasias em domicílio. Aspectos culturais e educacionais também podem orientar esse desfecho, visto que a decisão pessoal pode ser soberana na escolha do local de óbito.

Diante do exposto, visando avaliar o panorama epidemiológico e temporal dos óbitos por neoplasias malignas no Brasil, sobretudo no que se refere ao cenário de ocorrência para o momento da finitude da vida nas duas últimas décadas, definiu-se como objetivo deste estudo: analisar as características sociodemográficas e as tendências temporais dos óbitos por neoplasias malignas no Brasil, segundo a ocorrência em domicílio ou hospital, no período de 2002 a 2022.

## MÉTODOS

### Desenho e contexto

Estudo descritivo e ecológico que teve como unidades de análise os óbitos por neoplasias e as unidades da federação (UF) do Brasil. De acordo com dados censitários de 2022, o Brasil conta com uma população de 203.080.756 habitantes e área territorial de 8.510.417,771 km^2^, subdividindo-se em 26 estados e no Distrito Federal^21^. O produto interno bruto (PIB) *per capita* estimado para 2021 foi de R$ 42.247,52^21^, com índice de Gini variando em torno de 0,52 nesse mesmo ano^22^.

### Período e fonte de dados

Foram considerados os registros de óbito, entre 2002 e 2022, no Sistema de Informação sobre Mortalidade (SIM), acessado em 30 de abril de 2024 pelo Departamento de Informática do Sistema Único de Saúde (DATASUS). A definição do período de estudo (2002–2022) se justifica pelo fato de que, em 2002, foi instituído o Programa Nacional de Assistência à Dor e Cuidados Paliativos, e 2022 era o último ano com dados sobre mortalidade disponível no SIM na data de acesso ao DATASUS.

Complementarmente, foram considerados os dados da população brasileira, por meio do estudo de estimativas populacionais (por município, sexo e idade) referente ao período de 2000 a 2021, elaborado pelo Departamento de Análise Epidemiológica e Vigilância de Doenças Não Transmissíveis (DAENT) do Ministério da Saúde^23^. Em 2022, como os dados censitários ainda estavam indisponíveis no DATASUS, utilizou-se o quantitativo populacional estimado no estudo para 2021.

### População e critérios de seleção

Foram considerados os óbitos por neoplasias malignas de pessoas com idade igual ou superior a 20 anos com os códigos C00 a C97, da Classificação Estatística Internacional de Doenças e Problemas Relacionados à Saúde (10ª edição). Os dados foram acessados seguindo a UF da residência da pessoa e o ano de ocorrência do óbito. Foram excluídos os óbitos ocorridos em “outro estabelecimento de saúde”, “via pública” e “outros”, por não se tratar de locais de interesse ao estudo.

### Variáveis

Foram consideradas as variáveis: sexo (masculino; feminino; ignorado); faixa etária, em anos (20 a 29; 30 a 39; 40 a 49; 50 a 59; 60 a 69; 70 a 79; 80 ou mais); raça/cor (branca; preta; amarela; parda; indígena; ignorada); tempo de estudo, em anos (sem alfabetização; um a três; quatro a sete; oito a 11; 12 ou mais; ignorado); estado civil (solteiro; casado; viúvo; separado; outro; ignorado); e ano de óbito (2002 a 2022). Além disso, realizou-se a estratificação pela UF e regiões (Norte, Nordeste, Sul, Sudeste e Centro-Oeste).

### Tratamento e análise de dados

Inicialmente, as variáveis sociodemográficas foram descritas por meio de medidas da frequência absoluta (N/n) e relativa (%), segundo o local de ocorrência do óbito (domicílio; hospital). Como forma de comparar a diferença nas proporções entre as variáveis, foi aplicado o teste χ^2^ de Pearson, considerando o nível de significância de 5% (p<0,05). A análise dos resíduos ajustados serviu à direção de associação, sendo ela positiva (+) ou negativa (-) haja vista o valor superior a ±1,96^24^.

Também foram calculadas as taxas anuais de mortalidade, dividindo-se o número de óbitos pela população residente, no mesmo local e período, e multiplicando-se o resultado por 100 mil. Nas variáveis “sexo” e “faixa etária”, a população do denominador correspondeu àquela estimada com as mesmas características demográficas; para o local de residência, também se consideraram os dados estimados para a respectiva região/UF. Essas análises foram realizadas no Statistical Package for the Social Sciences Statistics, versão 25.

Com base nessas taxas, apresentaram-se os valores médios por triênios (2002–2004; 2005–2007; 2008–2010; 2011–2013; 2014–2016; 2017–2019; 2020–2022), permitindo-se a avaliação de sua mudança ao longo do período. Com as taxas anuais, realizou-se a análise de tendência por modelos do tipo *joinpoint regression* ^25^, estratificados pelas variáveis sociodemográficas e de residência, segundo o local de ocorrência. A análise foi realizada com auxílio do Joinpoint Regression Program, versão 5.0.2.

As taxas anuais de mortalidade por neoplasias, transformadas por função logarítmica natural (ln), foram consideradas como a variável dependente (y); e os anos-calendário do período, como a variável independente (x)^25^. Os modelos log-lineares [ln(y)=x’beta+erro], estimados via pesquisa em grade, foram ajustados pelos erros padrão dos coeficientes de mortalidade, calculados conforme as recomendações da literatura, e pela correção da autocorrelação de primeira ordem, verificada de acordo com os dados^25^.

Para os modelos finais, definidos pelo menor valor do critério de informação bayesiano ponderado, estimaram-se as variações percentuais anuais (VPA) e seus intervalos de confiança de 95% (IC95%). As VPA positivas/negativas indicaram a tendência crescente/decrescente das taxas de mortalidade, respectivamente, quando seus IC95% eram diferentes do valor nulo (0). As VPA cujo IC95% continham o valor nulo foram interpretadas como tendência estacionária^25^.

### Aspectos éticos

Nesta pesquisa foram utilizados apenas dados anônimos cujas informações disponíveis se encontram em formato agregado e sob domínio público, que podem ser acessados por qualquer cidadão/pesquisador pelo sítio eletrônico do DATASUS (https://datasus.saude.gov.br/). Portanto, conforme preconizado pela Resolução nº 674, de 6 de maio de 2022, do Conselho Nacional de Saúde do Brasil, não houve a necessidade de submissão nem de apreciação por Comitê de Ética em Pesquisa.

## RESULTADOS

Foram registrados 3.696.553 óbitos por neoplasias no Brasil. Na comparação entre os perfis de óbitos em domicílio e no hospital, observou-se associação significativa para todas as variáveis. Na análise dos resíduos ajustados, notou-se que foram positivamente associadas à ocorrência em domicílio as seguintes variáveis: sexo masculino; idade entre 70 e 79 anos e 80 anos ou mais; raça/cor parda e indígena; analfabetismo e tempo de estudo entre um e três anos; e estado civil viúvo ou outro (Tabela 1).

**Tabela 1:** Medidas descritivas das características sociodemográficas de pessoas com registro de óbito por neoplasias (CID-10: C00 a C97), segundo o local de ocorrência do óbito (domicílio; hospital), Brasil, 2002 a 2022.

Variável	Total		Domicílio		Hospital		Valor p*
	N	%	n	%	N	%	
**Sexo**							
Masculino	1.957.131	52,94	364.648^†^	56,46	1.592.483^‡^	52,20	<0,01
Feminino	1.739.190	47,05	281.231^‡^	43,54	1.457.959^†^	47,79	
Ignorado	232	0,01	26	0,00	206	0,01	
**Faixa etária (anos)**							
20 a 29	52.610	1,42	4.299^‡^	0,67	48.311^†^	1,58	<0,01
30 a 39	125.435	3,39	13.237^‡^	2,05	112.198^†^	3,68	
40 a 49	319.710	8,65	40.159^‡^	6,22	279.551^†^	9,16	
50 a 59	658.265	17,81	88.440^‡^	13,69	569.825^†^	18,68	
60 a 69	913.471	24,71	139.632^‡^	21,62	773.839^†^	25,37	
70 a 79	917.319	24,83	174.304^†^	26,99	743.015^‡^	24,36	
80 ou mais	709.743	19,20	185.834^†^	28,76	523.909^‡^	17,17	
**Raça/cor**							
Branca	2.193.085	59,33	346.580^‡^	53,65	1.846.505^†^	60,53	<0,01
Preta	256.208	6,93	42.727^‡^	6,62	213.481^†^	7,00	
Amarela	25.202	0,68	4.182^‡^	0,65	21.020^†^	0,69	
Parda	1.047.916	28,35	223.868^†^	34,66	824.048^‡^	27,01	
Indígena	5.240	0,14	1.475^†^	0,23	3.765^‡^	0,12	
Ignorada	168.902	4,57	27.073	4,19	141.829	4,65	
**Tempo de estudo (anos)**							
Nenhum	452.602	12,24	145.905^†^	22,59	306.697^‡^	10,05	<0,01
1 a 3	824.143	22,29	157.303^†^	24,35	666.840^‡^	21,86	
4 a 7	745.950	20,18	119.063^‡^	18,43	626.887^†^	20,55	
8 a 11	544.724	14,74	65.687^‡^	10,17	479.037^†^	15,70	
12 ou mais	297.626	8,05	35.041^‡^	5,43	262.585^†^	8,61	
Ignorado	831.508	22,50	122.906	19,03	708.602	23,23	
**Estado civil**							
Solteiro	710.014	19,21	123.415^‡^	19,11	586.599^†^	19,23	<0,01
Casado	1.697.139	45,91	288.704^‡^	44,69	1.408.435^†^	46,16	
Viúvo	747.045	20,21	152.242^†^	23,57	594.803^‡^	19,50	
Separado	258.057	6,98	33.957^‡^	5,26	224.100^†^	7,35	
Outro	81.201	2,20	16.304^†^	2,52	64.897^‡^	2,13	
Ignorado	203.097	5,49	31.283	4,84	171.814	5,63	
**Ano do óbito**							
2002–2004	379.148	10,26	73.526	11,38	305.622	10,02	<0,01
2005–2007	434.925	11,77	84.540	13,09	350.385	11,49	
2008–2010	485.961	13,15	87.113	13,49	398.848	13,07	
2011–2013	532.728	14,41	86.564	13,40	446.164	14,63	
2014–2016	582.551	15,76	89.474	13,85	493.077	16,16	
2017–2019	633.165	17,13	99.603	15,42	533.562	17,49	
2020–2022	648.075	17,52	125.085^‡^	19,37	522.990^†^	17,14	
**Total**	**3.696.553**	**100,00**	**645.905**	**100,00**	**3.050.648**	**100,00**	**NSA**

CID-10: Classificação Estatística Internacional de Doenças e Problemas Relacionados à Saúde (10ª edição); NSA: não se aplica;

*teste χ^2^ de Pearson (desconsiderados os casos ignorados);

^†^associação positivamente significativa;

^‡^associação negativamente significativa.

A série histórica dos óbitos ocorridos no período em estudo demonstra padrões distintos entre as variáveis. De modo geral, as taxas médias de mortalidade, no período, por neoplasias malignas no hospital foram superiores àquelas em domicílio, com destaque para as características: sexo masculino (87,19/100.000), idade de 80 anos ou mais (822,87/100.000), raça/cor branca (46,78/100.000), um a três anos de estudo (20,57/100.000) e estado civil casado (35,46/100.000) (Material Suplementar 1: https://doi.org/10.17632/fbcr95km4d.1 ).

As regiões Nordeste e Sul tiveram os maiores coeficientes de mortalidade em domicílio, chegando a 21,25/100.000 em 2022 e 20,64/100.000 em 2007, respectivamente. Em 2022, as maiores taxas médias de mortalidade no hospital foram observadas nas regiões Sudeste (79,72/100.000) e, novamente, Sul (90,26/100.000). Além disso, os estados com maiores valores foram Ceará (33,49/100.000), no domicílio, e Rio Grande do Sul (118,87/100.000), em hospital (Material Suplementar 2: https://doi.org/10.17632/fbcr95km4d.1 ).

Notou-se tendência crescente mais acentuada para os óbitos no domicílio entre homens e no hospital entre mulheres. No que se refere à faixa etária, a tendência foi majoritariamente estacionária. Quanto à raça/cor e ao estado civil, houve incrementos na série histórica de todos os óbitos em hospital. Em relação à escolaridade, observou-se aumento em todas as estratificações, exceto nas pessoas com de um a três anos de estudo que foram a óbito em hospital (Tabela 2).

**Tabela 2:** Tendência temporal das taxas brutas de mortalidade (a cada 100 mil habitantes) por neoplasias (CID-10: C00 a C97), segundo o local de ocorrência do óbito (domicílio; hospital), de acordo com as características sociodemográficas da pessoa, Brasil, 2002 a 2022.

Variável	Domicílio			Hospital		
	VPA	IC95%	Tendência	VPA	IC95%	Tendência
**Sexo**						
Masculino	1,72	0,76–2,79	Crescente	1,93	1,30–2,78	Crescente
Feminino	1,53	0,12–3,00	Crescente	2,54	2,00–3,25	Crescente
**Faixa etária (anos)**						
20 a 29	-1,49	-3,25–0,18	Estacionária	0,37	-0,02–0,77	Estacionária
30 a 39	-1,65	-2,54–-0,71	Decrescente	0,33	0,06–0,64	Crescente
40 a 49	-2,02	-3,22–-0,92	Decrescente	-0,67	-1,04–-0,29	Decrescente
50 a 59	-1,29	-2,40–-0,11	Decrescente	-0,58	-1,55–0,35	Estacionária
60 a 69	-0,88	-2,43–0,69	Estacionária	-0,00	-0,48–0,58	Estacionária
70 a 79	-0,74	-1,82–0,39	Estacionária	0,11	-0,56–0,92	Estacionária
80 ou mais	-0,57	-1,54–0,63	Estacionária	0,10	-0,71–1,19	Estacionária
**Raça/cor**						
Branca	0,90	-0,71–2,24	Estacionária	1,47	1,01–1,99	Crescente
Preta	3,11	1,82–4,42	Crescente	3,38	2,96–3,84	Crescente
Amarela	0,63	-0,65–2,08	Estacionária	2,05	1,65–2,47	Crescente
Parda	3,64	2,44–5,09	Crescente	4,96	3,86–6,49	Crescente
Indígena	1,25	0,87–1,56	Crescente	2,45	2,09–2,86	Crescente
**Tempo de estudo (anos)**						
Nenhum	1,41	0,34–2,52	Crescente	1,73	0,67–3,07	Crescente
1 a 3	1,10	0,66–1,55	Crescente	1,96	-1,60–4,35	Estacionária
4 a 7	4,06	2,59–5,52	Crescente	3,38	2,55–4,51	Crescente
8 a 11	7,59	6,21–9,32	Crescente	7,86	7,24–8,78	Crescente
12 ou mais	4,29	1,85–6,70	Crescente	4,78	4,30–5,36	Crescente
**Estado civil**						
Solteiro	2,12	0,67–3,74	Crescente	3,11	2,68–3,59	Crescente
Casado	0,34	-0,81–1,38	Estacionária	1,19	0,65–1,81	Crescente
Viúvo	1,49	0,37–2,50	Crescente	1,81	1,20–2,50	Crescente
Separado	6,50	5,25–8,47	Crescente	5,20	4,38–6,52	Crescente
Outro	7,76	4,71–13,69	Crescente	7,01	6,74–12,42	Crescente

CID-10: Classificação Estatística Internacional de Doenças e Problemas Relacionados à Saúde (10ª edição); VPA: variação percentual anual; IC95%: intervalo de confiança de 95% (limite inferior; limite superior).

Nacionalmente, teve-se tendência crescente tanto para os óbitos ocorridos em domicílio quanto para aqueles em hospital; o mesmo foi observado para as regiões Norte e Nordeste. Na avaliação dos óbitos ocorridos em domicílio, observou-se tendência de incremento em nove estados e no Distrito Federal, destacando-se a Bahia com o mais acentuado. No que se refere aos óbitos em hospital, todas as UFs registraram tendência crescente, com destaque para o Piauí (Tabela 3).

**Tabela 3:** Tendência temporal das taxas brutas de mortalidade (a cada 100 mil habitantes) por neoplasias (CID-10: C00 a C97), segundo o local de ocorrência do óbito (domicílio; hospital), de acordo com o local de residência da pessoa, Brasil, 2002 a 2022.

Variável	Domicílio			Hospital		
	VPA	IC95%	Tendência	VPA	IC95%	Tendência
**Região Norte**	1,88	1,15–2,75	Crescente	3,95	2,91–5,53	Crescente
Rondônia	2,49	0,02–5,64	Crescente	4,11	3,09–5,47	Crescente
Acre	1,90	-0,25–4,81	Estacionária	2,82	1,30–5,10	Crescente
Amazonas	1,63	0,83–2,63	Crescente	2,99	1,56–4,92	Crescente
Roraima	1,40	-0,64–4,39	Estacionária	3,41	2,36–4,86	Crescente
Pará	2,28	1,48–3,24	Crescente	4,38	3,61–5,46	Crescente
Amapá	-1,05	-4,02–2,43	Estacionária	4,50	2,83–7,32	Crescente
Tocantins	0,88	-0,94–2,95	Estacionária	4,60	3,13–6,86	Crescente
**Região Nordeste**	2,31	0,81–4,33	Crescente	4,02	3,20–5,33	Crescente
Maranhão	2,27	-0,53–6,70	Estacionária	5,28	4,10–7,30	Crescente
Piauí	2,09	0,54–4,11	Crescente	5,65	4,23–8,38	Crescente
Ceará	1,16	0,03–2,45	Crescente	4,05	3,13–5,39	Crescente
Rio Grande do Norte	1,18	-0,89–3,79	Estacionária	3,63	2,67–4,99	Crescente
Paraíba	1,09	-1,65–4,94	Estacionária	4,93	3,90–6,58	Crescente
Pernambuco	1,21	-0,09–2,75	Estacionária	2,61	1,87–3,54	Crescente
Alagoas	1,51	-0,24–3,59	Estacionária	5,18	4,39–6,26	Crescente
Sergipe	1,35	-0,11–2,96	Estacionária	3,02	1,67–4,86	Crescente
Bahia	4,43	3,02–6,37	Crescente	4,00	2,96–5,32	Crescente
**Região Sudeste**	1,18	-0,65–2,87	Estacionária	1,57	1,08–2,20	Crescente
Minas Gerais	2,68	1,15–4,40	Crescente	2,76	2,09–3,57	Crescente
Espírito Santo	-0,39	-2,13–1,39	Estacionária	2,43	1,49–3,64	Crescente
Rio de Janeiro	-0,09	-1,81–1,58	Estacionária	0,83	0,34–1,41	Crescente
São Paulo	1,07	-0,68–2,86	Estacionária	1,40	0,96–1,93	Crescente
**Região Sul**	1,20	-0,47–2,76	Estacionária	1,94	1,44–2,58	Crescente
Paraná	0,21	-0,91–1,36	Estacionária	2,34	1,70–3,09	Crescente
Santa Catarina	1,68	0,13–3,39	Crescente	2,59	1,97–3,38	Crescente
Rio Grande do Sul	1,98	-0,05–3,91	Estacionária	1,52	0,98–2,16	Crescente
**Região Centro-Oeste**	1,15	-0,20–2,72	Estacionária	2,48	1,80–3,36	Crescente
Mato Grosso do Sul	-0,41	-2,04–1,36	Estacionária	2,36	1,71–3,13	Crescente
Mato Grosso	0,66	-1,10–2,88	Estacionária	3,07	2,29–4,10	Crescente
Goiás	2,03	0,65–3,82	Crescente	2,71	1,77–3,97	Crescente
Distrito Federal	1,55	0,51–2,77	Crescente	1,38	0,80–2,10	Crescente
**Brasil**	1,58	0,46–2,83	Crescente	2,19	1,62–3,00	Crescente

CID-10: Classificação Estatística Internacional de Doenças e Problemas Relacionados à Saúde (10ª edição); VPA: variação percentual anual; IC95%: intervalo de confiança de 95% (limite inferior; limite superior).

## DISCUSSÃO

Com a análise de tendência estratificada, este estudo ofereceu resultados únicos e abrangentes de como características sociodemográficas influenciam o local de morte de pessoas com câncer ao longo de mais de 20 anos no Brasil, considerando também suas particularidades geoprogramáticas. Além disso, foram apresentadas as tendências estratificadas por aspectos sociodemográficos e territoriais, fornecendo *insights* de como o agravo tem acometido a população no decorrer do período avaliado.

A fim de contextualizar tais achados, estudos realizados em diferentes regiões do Brasil podem contribuir para a compreensão das tendências identificadas. No município de São Paulo (SP), características individuais como sexo feminino, raça/cor preta e parda, estado civil casado/viúvo e escolaridade elevada se associaram a menor chance de óbito domiciliar^17^. Embora os resultados sejam similares, há divergências com os resultados da presente pesquisa, em que ser viúvo se associou à ocorrência no domicílio.

Outro fator relevante que pode influenciar o local de morte é a idade avançada. Essa relação já foi verificada em estudos nacionais^14,17^ e internacionais^7,19,20,26^. As pessoas idosas em cuidados de fim de vida por causa do câncer avançado, em geral, são atendidas em casa por cuidadores, formais ou informais, o que pode contribuir, em grande parte, para que permaneçam nesse ambiente até o fim da vida^5,19^. Esse fenômeno pode se dar tanto por uma opção do próprio paciente quanto por decisão familiar.

Maior ocorrência de óbitos no domicílio em indivíduos do sexo masculino também já foi evidenciada^14,17,20^. Isso pode estar relacionado com o fato de a mulher tradicionalmente ter maior disposição para assumir o papel de cuidadora^17^. Embora não se possa estabelecer uma relação causal quanto a isso, infere-se que as mulheres poderiam assumir um papel mais proativo no cuidado do parceiro/familiar com câncer em domicílio, contribuindo para o aumento dos óbitos nesse ambiente entre esse público.

Já a relação entre ser viúvo e morrer em casa pode ser explicada pela ausência de companheiro que incentive a busca por atendimento médico ou decida por ela^2,19^. Corroborando essa hipótese, pesquisa chinesa identificou que residir com o cônjuge aumenta a probabilidade de morte hospitalar em 72%^27^. Logo, o suporte familiar pode contribuir para que a morte ocorra no domicílio^19^. Em contrapartida, a falta de disponibilidade de cuidadores familiares para a assistência paliativa no domicílio a dificulta^2,28^.

Estudo brasileiro identificou que, apesar da predominância de óbitos hospitalares, a maioria dos adultos com câncer preferia morrer no próprio lar^10^; pesquisas internacionais reforçam essa preferência^17,29^. Contudo, além de nem sempre existir concordância entre paciente e cuidador^10^, poucos pacientes que desejam morrer em casa têm essa condição atendida^2,30^. O local real da morte depende não apenas da situação clínica do paciente^2,30^, mas também do seu relacionamento com o cuidador principal^2^.

O nível socioeconômico pode exercer forte impacto sobre o local de morte por neoplasias. Em estados mais desenvolvidos, a mortalidade hospitalar manteve-se elevada, refletindo maior acesso a hospitais habilitados em oncologia. Já estados como Bahia e Pará apresentaram tendências crescentes de óbitos domiciliares, possivelmente associadas a barreiras geográficas e ao menor grau de instrução, fatores que podem reduzir a busca por internações e favorecer a permanência em casa na terminalidade.

Para mais, nota-se que a diversidade cultural, socioeconômica e de assistência de saúde entre os países acarreta maneiras distintas de lidar com questões relacionadas à morte e ao morrer^10,19^. Esse cenário também é uma realidade no Brasil, por causa do seu vasto território e da importante miscigenação cultural, social e econômica, justificando, em partes, as tendências mais acentuadas em determinados locais e estratos, bem como as diferentes taxas de mortalidade nos estados.

As tendências de mortalidade por neoplasias nos estados refletem desigualdades estruturais do sistema de saúde brasileiro. São Paulo e Rio Grande do Sul apresentaram altas taxas hospitalares, possivelmente associadas à maior oferta de unidades de referência oncológica. Já estados do norte e nordeste, como Maranhão e Piauí, registraram tendências crescentes de óbitos domiciliares, o que pode estar relacionado à baixa cobertura de serviços especializados e a dificuldades de acesso.

Na última década, houve um aumento considerável no número de instituições de cuidados paliativos no Brasil, passando de 92 serviços, em 2012, para 198, em 2020, o que corresponde a 0,94 serviços/milhão de habitantes^31,32,33^. Embora a taxa de cobertura e o alcance desses serviços no país ainda sejam incipientes, o crescimento no número de mortes domiciliares pode estar relacionado à melhoria no acesso aos cuidados paliativos decorrente do aumento do número de serviços disponibilizados^18^.

A Estratégia Saúde da Família, o Programa Melhor em Casa e a Política Nacional de Humanização também podem impulsionar a transição do óbito em hospital para domicílio^34,35,36^. Essas estratégias podem proporcionar maior acesso aos cuidados paliativos entre a população, bem como levar em consideração as singularidades e os desejos de cada indivíduo, favorecendo uma assistência que proporcione qualidade de vida aos dias que restam, de forma mais inclusiva e integral.

Todavia, estudo que analisou os desafios e as possibilidade do retorno de pacientes em cuidados paliativos para o domicílio constatou que, no Brasil, os recursos disponíveis pelo SUS para os cuidados no fim de vida no domicílio ainda são frágeis^35^. Esse dado precisa ser considerado na interpretação dos achados da presente pesquisa, visto que as maiores taxas de óbito por neoplasia maligna em domicílio podem decorrer da indisponibilidade de serviços hospitalares nos diferentes locais do país.

Há que se pontuar os efeitos da pandemia de COVID-19 nos resultados desta pesquisa, visto que ela influenciou as mortes por câncer e inúmeras outras causas em todo o mundo. No Japão, após 2019, considerando todas as causas, houve aumento do número de mortes em domicílio e diminuição das mortes hospitalares, especialmente entre pessoas com 65 anos ou mais^37^. Fatores como competição por leitos e a restrição no acesso aos serviços de saúde contribuíram para essa mudança^37,38^.

Embora esse não tenha sido o foco principal do presente estudo, tais achados podem explicar parcialmente os desta pesquisa durante o período de pandemia, contudo cumpre pontuar que, no tipo de abordagem adotada, séries históricas com intervalos longos, como as análises de cerca de 20 anos, tendem a ser menos sensíveis a flutuações pontuais em um curto intervalo de tempo, como as relacionadas aos efeitos específicos de um evento recente, a exemplo da pandemia.

Em âmbito nacional, a ausência de um programa que direcione corretamente os cuidados de fim de vida pode fazer com que as mortes em domicílio por câncer estejam relacionadas à dificuldade de acesso, e não a uma opção individual. A aprovação da Política Nacional de Cuidados Paliativos no âmbito do SUS, em 2023^34^, pode favorecer o desenvolvimento e a divulgação de materiais que tenham como propósito orientar os profissionais de saúde brasileiros em relação aos cuidados de fim de vida.

Portanto, os profissionais de saúde, sobretudo os enfermeiros, por sua maior proximidade com a população, precisam estar preparados para auxiliar as famílias e, até mesmo, as pessoas com câncer a refletirem sobre os aspectos positivos e negativos de morrer em casa ou no hospital. Entende-se que o apoio e aconselhamento de uma equipe multiprofissional são determinantes na garantia da autonomia e na consequente tomada de decisão da família/do paciente em relação aos cuidados de fim de vida.

As possíveis limitações do estudo decorrem da utilização de dados secundários, que podem conter dados incompletos, erros de preenchimento ou subnotificação de mortes por neoplasias malignas. Como são usadas fontes secundárias, não é possível afirmar se o local do óbito foi uma escolha do paciente. Outro fator que pode ter interferido nos resultados da pesquisa foi a pandemia de COVID-19, uma vez que ela influenciou as mortes por neoplasias e inúmeras outras causas, em nível global.

De qualquer maneira, os resultados deste estudo contribuem para uma compreensão mais ampla da relação entre os fatores sociodemográficos e o local de óbito de pacientes com câncer. Observou-se que houve aumento progressivo de mortes por câncer em hospitais, enquanto o crescimento dos óbitos em domicílio ocorreu apenas em algumas localidades e foi influenciado por fatores como sexo masculino, idade avançada, raça/cor parda ou indígena, baixa escolaridade (até três anos de estudo) e estado civil viúvo.

Destarte, os fatores que influenciam no local de morte de pessoas com câncer são multifacetados e podem estar relacionados com rede de apoio (in)existente, ao (in)acesso e à utilização dos serviços de saúde, ao contexto sociocultural e às características sociodemográficas. Em decorrência disso, fazem-se necessários estudos, incluindo com abordagem qualitativa, que explorem e aprofundem os aspectos que influenciam na escolha do local de morte de pessoas com câncer.

## Data Availability

Esta pesquisa utilizou dados anônimos e de domínio público cujas informações disponíveis se encontram em formato agregado. Portanto, conforme o preconizado pela Resolução nº 674, de 6 de maio de 2022, do Conselho Nacional de Saúde, dispensaram-se a submissão e apreciação por Comitê de Ética em Pesquisa.
